# Individual and synergistic protective properties of *Salvia officinalis* decoction extract and sulfasalazine against ethanol-induced gastric and small bowel injuries

**DOI:** 10.1039/d0ra03265d

**Published:** 2020-09-30

**Authors:** Saber Jedidi, Foued Aloui, Kais Rtibi, Houcem Sammari, Houcine Selmi, Ahmed Rejeb, Lamjed Toumi, Hichem Sebai

**Affiliations:** Unité de Physiologie Fonctionnelle et Valorisation des Bio-Ressources, Université de Jendouba, Institut Superieur de Biotechnologie de Beja Avenue Habib Bourguiba, B.P. 382 9000 Beja Tunisia sebaihichem@yahoo.fr +216 78 459 098 +216 97 249 486; Laboratoire des Ressources Sylvo-Pastorales, Université de Jendouba, Institut Sylvo-Pastoral de Tabarka B.P. 345 8110 Tabarka Tunisia; Universite de Carthage, Faculté des Sciences de Bizerte 7021 Jarzouna Tunisia; Laboratoire d'Anatomie Pathologique, Université de Manouba, Ecole Nationale de Médecine Vétérinaire de Sidi Thabet 2020 Sidi Thabet Tunisia

## Abstract

The present study was carried out to determine the phytochemical composition of *Salvia officinalis* flowers decoction extract (SOFDE) as well as its individual and/or synergistic actions with sulfasalazine against ethanol (EtOH)-induced peptic ulcer in Wistar rats. In this respect, rats were divided into six groups of eight animals each: control, EtOH, EtOH + sulfasalazine (SULF, 100 mg kg^−1^, b.w., p.o.), mixture: MIX (SOFDE, 50 mg kg^−1^ b.w., p.o. + SULF, 50 mg kg^−1^, b.w., p.o.) and EtOH + two doses of SOFDE (100 and 200 mg kg^−1^ b.w., p.o.). *In vitro*, the phytochemical and the antioxidant properties were determined using colorimetric analysis. HPLC-PDA/ESI-MS assay was used to identify the distinctive qualitative profile of phenolic compounds. Our results firstly indicated that SOFDE is rich in total tannins, flavonols, anthocyanins and a moderate concentration of total carotenoids. Chromatographic techniques allowed the identification of 13 phenolic compounds and the major ones are quinic acid, protocatechuic acid, gallic acid and salviolinic acid. SOFDE also exhibited an important *in vitro* antioxidant activity using the β-carotene bleaching method. *In vivo*, SOFDE and the mixture provide significant protection against ethanol-induced gastric and duodenal macroscopic and histological alterations. Also, SOFDE alone or in combination with SULF, showed a significant protection against the secretory profile disturbances, lipid peroxidation, antioxidant enzyme activities and non-enzymatic antioxidant level depletion induced by alcohol administration. Importantly, we showed that EtOH acute intoxication increased gastric and intestinal calcium, free iron, magnesium and hydrogen peroxide (H_2_O_2_) levels, while SOFDE/MIX treatment protected against all these intracellular mediators' deregulation. We also showed that alcohol treatment significantly increased the C-reactive protein (CRP) and alkaline phosphatase (ALP) activities in plasma. The SOFDE and MIX treatment significantly protected against alcohol-induced inflammation. More importantly, we showed in the present work that the mixture exerted a more important effect than SOFDE and SULF each alone indicating a possible synergism between these two molecules. In conclusion, our data suggests that SOFDE and SULF exerted a potential synergistic protective effect against all the macroscopic, histological and biochemical disturbances induced by EtOH intoxication. This protection might be related in part to its antioxidant and anti-inflammatory properties as well as by negatively regulating Fenton reaction components such as H_2_O_2_ and free iron.

## Abstract 2

The present study was carried out to determine the phytochemical composition of *Salvia officinalis* flowers decoction extract (SOFDE) as well as its individual and/or synergistic actions with sulfasalazine against ethanol (EtOH)-induced peptic ulcer in Wistar rats.

## Graphical abstract

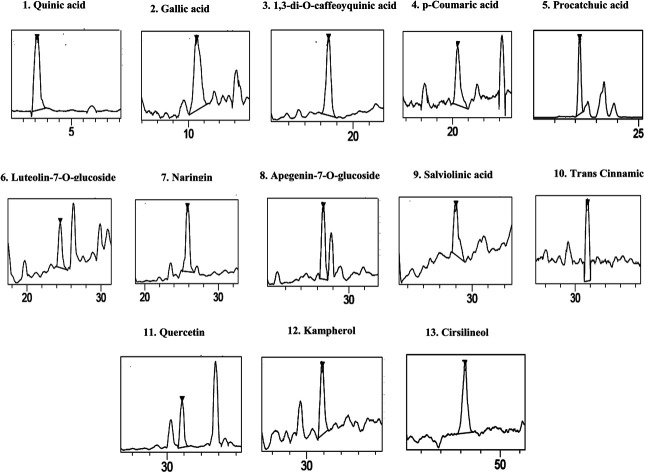

## Introduction

Peptic ulcer is caused by the loss of balance between aggressive and defensive factors of the gastric and duodenal mucosa.^1^ Also, it can be caused by *Helicobacter pylori*,^2^ the use of non steroidal anti-inflammatory drugs,^3^ smoking,^4^ the imbalance between the secretion of hydrochloric acid as well as the production of calcium bicarbonate to buffer the pH in the gastrointestinal microbiota.^5^

Alcohol consumption is a well-known risk factor for tissue injury and gastroduodenal ulcer. It also affects other organs such as the heart, kidneys, brain, liver and pancreas.^6,7^

Moreover, previous studies have shown an inhibitory effect on the synthesis of prostaglandins leading to lesions of the gastric mucosa. This disease may also be related to neutrophil activation leading to an excessive production of reactive oxygen species (ROS).^8^ Under physiological conditions, ROS are produced in small quantities during cellular respiration and metabolism, which are important for several physiological processes.^9,10^ However, the intracellular imbalance between their genesis and degradation contributes to oxidative stress. This situation has been accompanied by significant damage of lipids, proteins and nucleic acids leading to cell death.^11,12^

The mixture of bioactive compounds from plant and synthetic drugs is an alternative for the protection and/or treatment of various pathologies and to substitute commercial drugs known for their unpredictable side effects.^13^ However, in the gastrointestinal system, prolonged use of drugs (anticholinergic drugs, histamine H2-receptor antagonists, antacids) and especially with relatively high doses can exhibit toxic side effects leading to severe constipation, diarrhea, hypersensitivity reactions such as rashes, fever, central nervous system aberrations^14^ and colorectal cancer.^15^ In women, misoprostol can cause malformations of the embryo.^16^ Importantly, we recently used a mixture between sage and loperamide as a strategy to fight against castor oil-induced diarrhea.^17^

*Salvia officinalis* (Lamiaceae family) is known as a medicinal and aromatic plant due to its richness of natural active substances.^18^ Due to its antioxidant^19^ and anti-inflammatory^20^ properties, sage extracts exhibit many beneficial health effects such as phytoestrogenic,^21^ neurprotective,^22^ anti-microbial^23^ and anticancer^24^ activities. More importantly, this plant has been widely used in the treatment of most gastrointestinal diseases like diarrhea and dyspepsia.^17,25^

*Salvia officinalis* is an inexhaustible reservoir of chemical compounds such as alkaloids, carbohydrate, fatty acids, glycosidic derivatives (flavonoid glycosides, saponins), phenolic compounds (coumarins, flavonoids, tannins), polyacetylenes, steroids, terpenes (monoterpenes, diterpenes, triterpenoids).^26,27^ However, Mansourabadi *et al.*^28^ reported that flavonoids extracted from *Salvia officinalis* presented anti-inflammatory properties in carrageenan model of mouse and induced analgesic effect in a dose-dependent manner. In addition, several others molecules such as manool, carnosol and ursolic acid have been previously showed for their anti-inflammatory properties.^29,30^

Hence, the present study aimed to investigate the individual and synergistic protective properties of *Salvia officinalis* flowers decoction extract and sulfasalazine against ethanol-induced peptic ulcer in rat.

## Experimental

### Reagents and chemicals

2-Thio-barbituric acid (TBA), epinephrine, bovine catalase, trichloroacetic acid (TCA), butylated hydroxyl toluene (BHT) were from Sigma chemicals Co. (Sigma-Aldrich GmbH, Steinheim, Germany). Ethanol (EtOH), sulfasalazine, sodium chloride (NaCl 0.9%) were purchased from central pharmacy of Tunisia. All the other chemicals used were of analytical grade.

### Plant material and decoction extract

The sage flowers were cultivated in the region of Ain Draham (NW-Tunisia) during April 2018 and identified by Dr Imen Bel Hadj Ali, Associate professor in the Higher Institute of Biotechnology of Béja-Tunisia. The voucher specimens (No. SO.321) have been deposited with the Herbarium of the Higher Institute of Biotechnology of Béja. The plant material was dried in the open air and powdered in an electric blender. The decoction was made with distilled water (1/5; w/v) at 100 °C during five minutes under magnetic agitation. The homogenate was filtered by Whatman filter papers and was evaporated at 40 °C in a ventilated oven.

### Phytochemical properties and antioxidant capacity

#### Mineral determination

One gram of powder was placed in a muffle furnace for calcination process (Tony Güller Orselina Zürich MOD L 51/5) at 550 °C for 4 hours.^31^ Then the magnesium, iron and calcium concentrations in the samples were determined by an atomic absorption flame spectrophotometer (SHIMADZU AA-6200).

#### Identification of phenolic compounds by liquid chromatography-high resolution electrospray ionization mass spectrometry (LC-HRESIMS) assay

The analysis for phenolic compounds was performed on a Shimadzu UFLC XR system (Kyoto, Japan), equipped with a SIL-20AXR auto-sampler, a CTO-20 AC column oven, a LC-20ADXR binary pump and a quadripole 2020 detector system. Briefly, 100 mg of the plant extract (SOFDE) were dissolved in 100 mL of 10% methanol and filtered and then 1 mL was transferred into LC-MS vials. An opposite-phase column (Pursuit XRs ULTRA 2.8, C18, 100 × 2 mm, Agilent Technologies, UK) was used to carry out HPLC investigations. 20 μL of the prepared samples were injected at a column temperature set at 30 °C. Mobile phases consisted of 0.1% formic acid in water (A) and 0.1% formic acid in methanol (B). A gradient program was used for isolation at a flow rate of 1 mL min^−1^. Mobile phases consisted of an initial composition of 100% solvent A, with a gradient of 100% solvent B over 20 minutes, held at 100% solvent B for 5 min and 100% solvent A for 25 min. The drying gas flow rate was 1 mL min^−1^ at 320 °C. MS was operated in the positive ion mode in a mass range of 100–2000 *m*/*z*. High resolution mass spectral data were obtained on a Thermo Instruments ESI-MS system (LTQ XL/LTQ Orbitrap Discovery, UK) connected to a Thermo Instruments HPLC system (Accela PDA Detector, Accela PDA Autosampler and Accela Pump).^32^

#### Total tannins, flavonols, total carotenoids and total anthocyanins in SOFDE

Total tannins were evaluated by the method using the Folin-Ciocalteu reagent.^33^ In fact, 500 μL of Folin-Ciocalteu (50%) was added to 500 μL of extract followed by 1 mL of Na_2_CO_3_ (20%). Absorbance of the supernatant was measured using an ultraviolet (UV)-visible spectrophotometer (DU 640B, Beckman Coulter) at 730 nm.

The quantification of flavonols was determined as previously described by Rigane *et al.*^34^ Briefly, 1 mL AlCl_3_ (20%) was added to 1 ml of the extract and 3 mL of sodium acetate (50 mg mL^−1^). After incubation for 2 hours and 30 minutes, the absorbance was read at 440 nm. Results were expressed as mg of rutin equivalents per 100 gram of dry matter (mg RE/100 g DM).

Total carotenoids in SOFDE was evaluated by the procedure described by Marina *et al.*^35^ Firstly, 1 mL of the extract was mixed with 1 mL of distilled water and 2 mL of extraction solvent (hexane/acetone/ethanol; 50/25/25%; v/v/v) and the mixture was vortexed for 1 min and centrifuged at 6000*g* at 5 °C for 10 min. The upper layer of hexane containing the pigments was recovered and expelled to a 25 mL volumetric flask. The remaining layer was subjected to a second extraction (same procedure as the extract) and the hexane layers were combined and adjusted to 25 mL. The total carotenoid content was evaluated by adding 1 mL of hexane extract by measuring the absorbance at 450 nm and was expressed in β-carotene using the absorbance coefficient of 2500 according to the following formula:Total carotenoids (μg mL^−1^) = A450 × volume (mL) × 1000/2500 × weight of the sample (g)

Total anthocyanin compounds were evaluated according to the differentiation and using two buffers: KCl at pH 1.0 (0.025 M) and CH_3_COONa (0.025 M) pH 4.5 (0.4 M). 400 μL of the extract were mixed with 3.6 mL of the buffer (1), followed by 400 μL of the other, in buffer (2). The reaction mixture was incubated during 30 minutes in the dark and the absorbances are then read at 510 and 700 nm. The concentration of anthocyanin pigment in the extract is expressed as mg cyanidine equivalent glucosyl-3/g dry matter (mg ECy/g DM).^36^

#### Antioxidant activity by the β-carotene bleaching inhibition method

The antioxidant activity was performed by the method of β-carotene bleaching inhibition according to Kulisica *et al.*^37^ Indeed, the discoloration of β-carotene can be slowed down in the presence of antioxidant, which blocks the formation of free radicals. 0.2 mg of β-carotene, 20 mg of linoleic acid and 200 mg of tween 40 are dissolved in 0.5 mL of chloroform. The solvent was then evaporated and the mixture obtained was diluted with 50 mL of water bubbled with oxygen. 4 mL of the obtained emulsion was expelled into tubes respectively containing 0.2 mL of the extract or studied molecule, 0.2 mL of BHT: synthetic antioxidant for the comparative test and 0.2 mL of the solvent used which will serve as a negative witness. The blocked tubes were kept out of the light and at 50 °C in a water bath. The absorbance of the samples is measured at 470 nm at initial time and every 15 minutes during 120 minutes. The blank test is an emulsion prepared as above but without β-carotene. The coefficient of antioxidant activity (CAA) is determined by the following expression:AA% = 100 × [(*A*_E(0min)_ − *A*_E(120min)_)/(*A*_0(0min)_ − *A*_0(120min)_)]with *A*_0(0min)_, *A*_0(120min)_: absorbance of the control at *t* = 0 and *t* = 120 min, respectively;

*A*_E(0min)_ et *A*_E(120 min)_: absorbance of the sample analyzed at *t* = 0 and *t* = 120 min, respectively.

#### Animals and treatment

Adult male Wistar rats (weighing 214.97 ± 16.12 g) were purchased from the Society of Pharmaceutical Industries of Tunisia (SIPHAT, Ben-Arours, TN). All animal procedures were performed in accordance with the Guidelines for Care and Use of Laboratory Animals of Tunis University and approved by the Animal Ethics Committee of National Institute of Health. The test was performed in compliance with the Commission Directive 2000/32/EC and the OECD Guideline 474. They were provided with standard food (BADR, Utique, TN) and water ad libitum and maintained in animal house under controlled temperature (22 ± 2 °C) with a 12/12 h light-dark cycle.

Animals were divided into six groups of eight animals each. Group 1 and 2 served as controls and received distilled water (10 mL kg^−1^, b.w., p.o.) for 15 days. Groups 3 and 4 were pre-treated with various doses of the SOFDE (100, 200 mg kg^−1^, b.w., p.o.), group 5 received sulfasalazine (100 mg kg^−1^, b.w., p.o.), while group 6 was pre-treated with the mixture (MIX: SOFDE, 50 mg kg^−1^ b.w., p.o. + sulfasalazine, 50 mg kg^−1^, b.w., p.o.). However, preliminary experiment indicated that the selected doses present the lowest that gives significant protective effects. Rats were fasted for 18 h before the last administration of SOFDE, MIX or reference molecules. After 60 min, each animal, except group 1, was received EtOH (4 g kg^−1^, b.w.) by oral administration. Two hours later, rats were anaesthetized by intraperitoneal administration of sodium pentobarbital (40 mg kg^−1^, b.w.) and sacrificed by decapitation,^38^ blood was collected and plasma processed for electrolytes (free iron, calcium and magnesium), alkaline phosphatase (ALP) and C-reactive protein (CRP) determinations.

#### Gastric and intestinal fluid accumulation

The gastric and intestinal fluid was evaluated according to Dicarlo *et al.*^39^ The fluid was collected and centrifuged at 3000 g during 5 min to eliminate insoluble materials. The supernatant was after measured using graduate tubes. After weighting the stomach and the small intestine, the difference between full and empty of two organs were determined.

#### Evaluation of gastric and intestinal mucosal damage

The stomach and small intestine of each animal was thrown out and opened along its greater curvature. The tissue was gently rinsed in NaCl 0.9%. The lesions in the gastric mucosa were macroscopically examined and the photographs of hemorrhagic erosions were taken by Canon EOS1100 D (ISO 6400) digital camera. Ulcer indexes were determined as the sum of the lengths of the whole gastric lesions (mm^2^). Two independent, blinded observers performed the measurements of lesion lengths.

#### Histopathological analysis

Immediately after sacrifice, small pieces of stomach and duodenum were collected and washed with NaCl (0.9%). Tissue fragments were then fixed in a 10% neutral buffered formalin solution, embedded in paraffin and used for histopathological examination. 5 μm thick sections were cut, deparaffinized, hydrated and stained with hematoxylin and eosin (HE). The gastric and small intestines sections were examined in blind fashion in all treatments.

#### Plasma scavenging activities

The plasma scavenging activity (PSA) in the different groups was measured using the DPPH radical according the method of Brand-Williams *et al.*^40^ Briefly, 100 μL of plasma sample was added to 2 mL of 2,2-diphenyl-1-picrylhydrazyl (DPPH) in methanol solution (100 mM). 1 mL of chloroform was added after incubation of the solution at 37 °C for 30 min and the mixture was centrifuged at 3000 *g* during 10 min. The absorbance of clear supernatant was then determined at 517 nm. DPPH solution was used as a control and the PSA, expressed as percentage, was calculated according to the following equation:PSA (%) = 100 × (*A*_517_ (control) × *A*_517_ (sample)/*A*_517_ (control)).

#### Lipid peroxidation measurement

Gastric and duodenal mucosa lipid peroxidation was evaluated by MDA measurement according to the double heating method.^41^ Briefly, aliquots from stomach and duodenum mucosa homogenates were mixed with BHT–trichloroacetic acid (TCA) solution containing 1% BHT (w/v) dissolved in 20% TCA (w/v) and centrifuged at 1000 g for 5 min at 4 °C. Supernatant was blended with a solution containing (0.5 N HCl, 120 mM TBA buffered in 26 mM Tris) and then heated at 80 °C for 10 min. After cooling, the absorbance of the resulting chromophore was determined at 532 nm. MDA levels were calculated using an extinction coefficient for MDA–TBA complex of 1.56 × 105 M^−1^ cm^−1^.

#### H_2_O_2_ determination

The gastric and intestinal mucosa H_2_O_2_ level was determined according to Dingeon *et al.*^42^ However, the hydrogen peroxide reacts with *p*-hydroxybenzoic acid and 4-aminoantipyrine in the presence of peroxidase leading to the formation of quinoneimine that has a pink color detected at 505 nm.

#### Antioxidant enzyme activity assays

SOD activity in the gastric and duodenal mucosa was determined using modified epinephrine assays.^43^ At alkaline pH, superoxide anion induces the autoxidation of epinephrine to adenochrome; while competing with this reaction, SOD decreased the adenochrome formation. One unit of SOD is defined as the amount of the extract that inhibits the rate of adenochrome formation by 50%. Enzyme extract was added to 2 mL reaction mixture containing 10 μL of bovine catalase (CAT, 0.4 U mL^−1^), 20 μL of epinephrine (5 mg mL^−1^) and 62.5 mM of sodium carbonate/bicarbonate buffer (pH 10.2). Changes in absorbance were assessed at 480 nm.

The activity of catalase was recorded by measuring the initial rate of H_2_O_2_ disappearance at 240 nm.^44^ The reaction mixture contained 33 mM H_2_O_2_ in 50 mM phosphate buffer (pH 7) and the CAT activity was calculated using the extinction coefficient of 40 mM^−1^ cm^−1^ for H_2_O_2_.

The activity of glutathione peroxidase was quantified following the procedure of Flohé and Gunzler.^45^ Briefly, 1 mL of reaction mixture containing 0.2 mL of gastric or intestinal mucosa supernatant, 0.2 mL of phosphate buffer 0.1 M pH 7.4, 0.2 mL of GSH (4 mM) and 0.4 mL of H_2_O_2_ (5 mM) was incubated at 37 °C for 1 min and the reaction was stopped by the addition of 0.5 mL TCA (5%, w/v). After centrifugation at 1500*g* for 5 min, aliquot (0.2 mL) from supernatant was combined with 0.5 mL of phosphate buffer 0.1 M pH 7.4 and 0.5 mL DTNB (10 mM) and absorbance was read at 412 nm. The GPx activity was expressed as nM of GSH consumed/min/mg protein.

#### Non-enzymatic antioxidants levels

The total concentrations of thiol (–SH) groups in the gastric and intestinal mucosa were determined by Ellman's method.^46^ Aliquots of gastric or duodenal mucosa were mixed with 800 μL of 0.25 M phosphate buffer (pH 8.2) and 100 μL of 20 mM EDTA, and the optical density was measured at 412 nm (A1). Subsequently, we added 100 μL of 10 mM DTNB and the reaction mixture was incubated at 37 °C during 15 minutes and a new value (A2) was determined. The thiol groups concentration was calculated by the difference between A2 and A1 using a molar extinction coefficient of 13.6 × 10^3^ M^−1^ cm^−1^. The results are expressed in nM of thiol groups per mg of protein.

The level of GSH was performed by colorimetric method using the method of Sedlak and Lindsay.^47^ In fact, 5 mL of supernatant was mixed with 4 mL of cold distilled water and 1 mL of TCA (50%). The tubes were vortexed for 10 minutes and centrifuged at 1200 g for 15 minutes. 2 mL supernatant was mixed with 4 mL of 0.4 M Tris buffer (pH 8.9). 0.1 mL of DTNB (0.01 M) were added to the reaction medium. The absorbance was recorded rapidly at 412 nm against the blank containing only the buffer.

#### Protein determination

Protein concentration was determined according to Hartree, which is a slight change of the Lowry method. Serum albumin was used as a standard.^48^

#### Iron measurement, calcium and magnesium determination

Free iron, calcium and magnesium concentrations were performed using commercially available diagnostic kits (Biomaghreb, Ariana, TN, ISO 9001 certificate).

#### Quantitative determination of C-reactive protein (CRP) and ALP activity

Alkaline phosphatase activity and C-reactive protein content were assessed using commercially available diagnostic kits (Biomaghreb, Ariana, TN, ISO 9001 certificate).

#### Statistical analysis

The data were analyzed by one-way analysis of variance (ANOVA) and expressed as means ± standard error of the mean (S.E.M.). All analyzes were performed using the SAS (Statistics Analysis System). All statistical tests were two-tailed, and a *P* value of 0.05 or less was considered significant.

## Results

### Phytochemical composition of *Salvia officinalis* flowers decoction extract (SOFDE) and *in vitro* antioxidant capacity

#### Colorimetric and chromatographic analysis of SOFDE

We firstly showed that calcium is the most abundant elements in sage flowers extract while iron comes in last place (6.64 ± 0.04 mmol L^−1^ and 3.29 ± 0.08 μmol L^−1^, respectively). The SOFDE (extraction yield = 17.22 ± 0.13%) also contains a high content of total tannins (60.41 ± 3.87 TAE/g DM), flavonols (1.99 ± 0.02 mg RE/g DM) and anthocyanins (3.79 ± 0.29 mg CG/g DM), but a moderate concentration of total carotenoids (1.67 ± 0.09 μg/100 mL) (Table 1).

**Table 1:** Phytochemical composition and IC_50_ values of the β-carotene bleaching inhibition and chelating effect of the *Salvia officinalis* flowers decoction extract (SOFDE) and butylated hydroxytoluene (BHT)^a^

Parameters	Contents
Iron (μmol L^−1^)	3.29 ± 0.08
Magnesium (mmol L^−1^)	4.25 ± 0.02
Extraction yield (%)	17.22 ± 0.13
Calcium (mmol L^−1^)	6.64 ± 0.04
Total tannins (mg TAE/g DM)	60.41 ± 3.87
Flavonols (mg RE/g DM)	1.99 ± 0.02
Total carotenoids (μg/100 mL)	1.67 ± 0.09
Total anthocyanins content (mg CG/g DM)	3.79 ± 0.29
β-Carotene bleaching inhibition IC_50_ (μg mL^−1^)	56.77 ± 2.34
Butylated hydroxytoluene IC_50_ (μg mL^−1^)	20.83 ± 0.71

a Data are expressed as mean ± SEM (*n* = 3); SEM: standard error of the mean; DM: dry matter; TAE: tannic acid equivalent; CG: cyanidine glucosyl-3; RE: rutin equivalent.

**Fig. 1:**
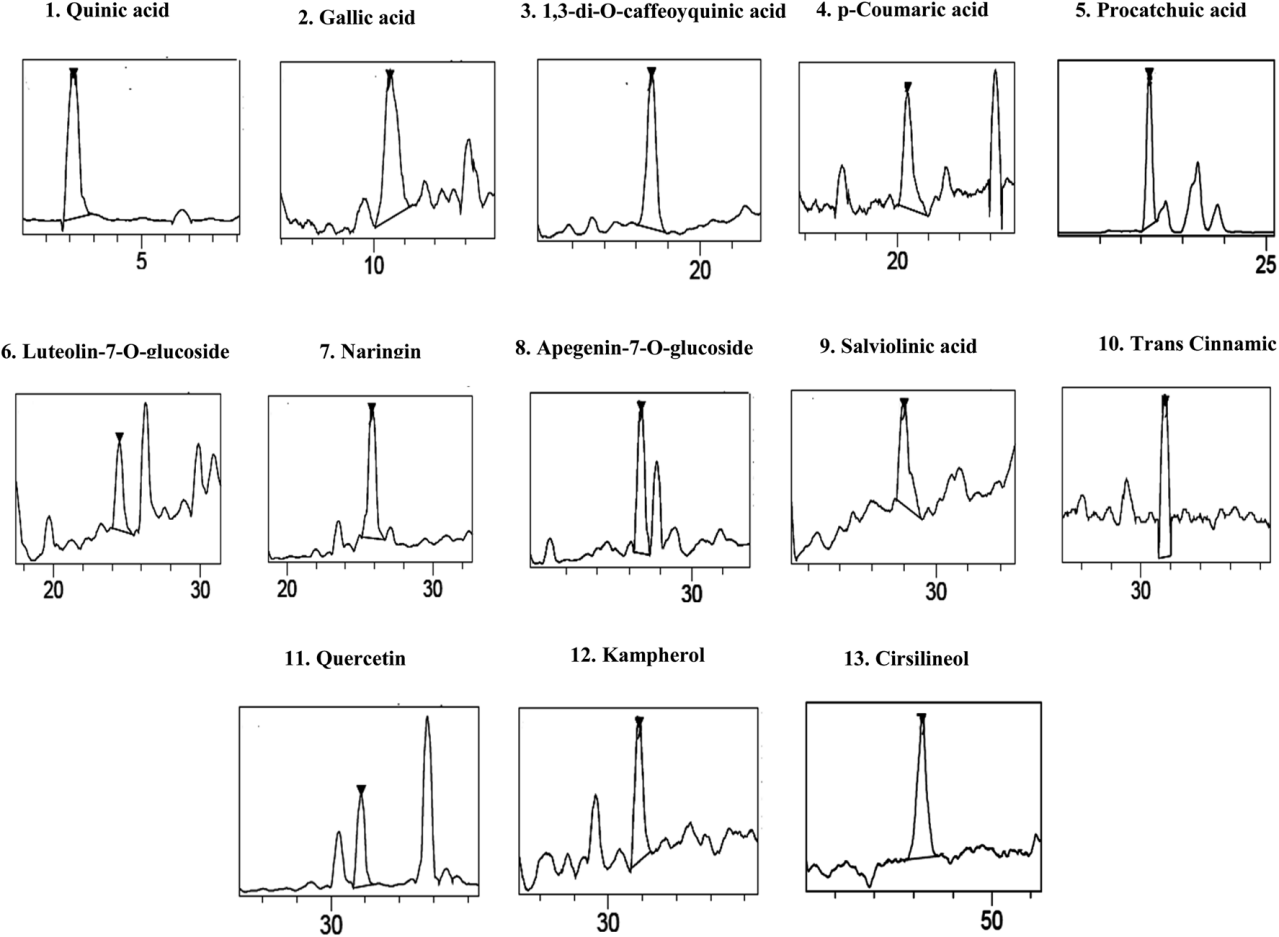
Chromatographic profile and characterization of phenolic compounds of *Salvia officinalis* L. flowers decoction extract (SOFDE) (assignments of peaks are given in Table 2).

The HPLCPDAESI-MS/MSLC-HRESIMS analysis of SOFDE allowed to the identification of 13 phenolic compounds. Six phenolic acids which include quinic acid, protocatechuic acid, gallic acid, 1,3-di-*O*-caffeoyquinic acid, *p*-coumaric acid and salviolinic acid (Table 2). In addition, the chromatographic elution profile of flavonoids (Fig. 1) showed seven flavonoids compounds such as naringin, quercetin, kampherol, apegenin-7-*o*-glucoside, *trans* cinnamic, luteolin-7-*O*-glucoside and cirsilineol.

**Table 2:** Liquid chromatography-high resolution electrospray ionization mass spectrometry (LC-HRESIMS) analysis of *Salvia officinalis* flowers decoction extract

Peak no.	Identification^a^	Formula	[M]^−^ H *m*/*z*^b^	Retention time (min)	Concentration (ppm)
1	Quinic acid	C_7_H_12_O_6_	191.00	2.142	181.883
2	Gallic acid	C_7_H_6_O_5_	169.00	3.986	158.708
9	1,3-Di-*O*-caffeoylquinic acid	ýC_25_H_24_O_12_	515.00	16.761	6.433
3	Protocatchuic acid	C_7_H_6_O_4_	153.00	6.825	155.249
11	*p*-Coumaric acid	C_9_H_8_O_3_	163.00	20.642	279.886
16	Luteolin-7-*O*-glucoside	C_21_H_20_O_11_	447.00	59.969	48.608
20	Naringin	C_27_H_32_O_14_	579.00	25.560	16.987
21	Apegenin-7-*O*-glucoside	C_21_H_20_O_10_	431.00	67.712	14.217
23	Salviolinic acid	C_36_H_30_O_16_	717.00	28.004	268.318
24	*Trans* cinnamic	C_9_H_8_0_2_	147.00	31.617	733.142
25	Quercetin	C_21_H_20_O_11_	301.00	31.740	40.272
26	Kampherol	C_15_H_10_O_6_	285.00	31.840	1.708
31	Cirsilineol	C_18_H_16_O_7_	343.00	38.998	5.271

a The compounds are suggested according to the dictionary of natural products and the characteristic fragmentation pattern.

b The formulae were deduced from the quasi molecular ion peak [M + H]^+^.

#### *In vitro* antioxidant capacity of SOFDE

Concerning the antioxidant capacity, we showed in Table 1 that the inhibition of β-carotene bleaching effect of SOFDE and Butylated hydroxyl toluene (BHT) increased significantly in a dose-dependent manner. The inhibit rice concentration 50 of SOFDE (IC_50_ = 56.77 ± 2.34 μg mL^−1^) appear significantly higher than BHT (IC_50_ = 20.83 ± 0.71 μg mL^−1^) used as reference molecule.

#### Evaluation of gastric and small bowel fluid accumulation

As shown in Tables 3 and 4, gastric and duodenal ulceration was accompanied by a significant increase in fluid accumulation as well as a decrease in mucus weights. The administration of SOFDE and MIX has been significantly restored, all of these parameters in a dose-dependent manner. We found the most relevant correction was recorded in the group that was received a mixture dose of SOFDE and sulfasalazine. In addition, sulfasalazine was also protected against disruption of the secretory profile.

**Table 3:** Evaluation of gastric and small bowel fluid accumulation. Animals were pre-treated with two doses of SOFDE (100 and 200 mg kg^−1^, b.w., p.o.), mixture (SOFDE, 50 mg kg^−1^, b.w., p.o. + SULF, 50 mg kg^−1^, b.w., p.o.) and sulfasalazine (100 mg kg^−1^, b.w., p.o.) or vehicle (NaCl 0.9%). One hour after, animals received EtOH (4 g kg^−1^, b.w., p.o.) by gavage for 2 h. Assays were carried out in triplicate^a^

Groups	Weight of stomach content (g)	Protection (%)	Weight of intestinal content (g)	Protection (%)
Control	0.51 ± 0.18		0.34 ± 0.37	
EtOH	5.39 ± 1.36*	0	2.13 ± 0.90*	0
EtOH + SOFDE-100	4.57 ± 1.22#	15.21	0.98 ± 0.72#	53.99
EtOH + SOFDE-200	4.08 ± 0.87#	24.30	0.75 ± 0.30#	64.79
EtOH + SULF	3.85 ± 1.43#	41.56	0.56 ± 0.12#	73.71
EtOH + MIX	2.99 ± 1.03#	59.37	0.39 ± 0.09#	81.69

a *: *p* < 0.05 compared to control group and #: *p* < 0.05 compared to EtOH group.

**Table 4:** Effects of *Salvia officinalis* flowers decoction extract (SOFDE), mixture (MIX) and sulfasalazine (SULF) on EtOH-induced acute macroscopic gastric and small bowel injury. Animals were pre-treated with two doses of SOFDE (100 and 200 mg kg^−1^, b.w., p.o.), mixture (SOFDE, 50 mg kg^−1^, b.w., p.o. + SULF, 50 mg kg^−1^, b.w., p.o.) and SULF (100 mg kg^−1^, b.w., p.o.) or vehicle (NaCl 0.9%). One hour after, animals received EtOH (4 g kg^−1^, b.w., p.o.) by gavage for 2 h. Assays were carried out in triplicate^a^

Groups	Mucus weight (g)	Protection (%)	Ulcer index (mm^2^)	Protection (%)	Small bowel injury index (mm^2^)	Protection (%)
Control	4.53 ± 0.57		0			
EtOH	1.74 ± 0.56*	0	73.60 ± 4.05*	0	20.82 ± 3.09*	
EtOH + SOLAE-100	2.62 ± 0.98#	50.57	51.48 ± 3.83#	30.05	13.34 ± 4.01#	35.93
EtOH + SOLAE-200	2.95 ± 0.46#	69.54	39.80 ± 1.50#	45.92	10.26 ± 1.37#	50.72
EtOH + SULF	2.82 ± 0.26#	62.07	29.75 ± 0.26#	59.58	7.41 ± 0.36#	64.41
EtOH + MIX	3.02 ± 0.19#	73.56	10.13 ± 2.36#	86.24	6.94 ± 0.43#	66.67

a *: *p* < 0.05 compared to control group and ^#^: *p* < 0.05 compared to EtOH group.

#### Qualitative and quantitative macroscopic evaluation

**Fig. 2:**
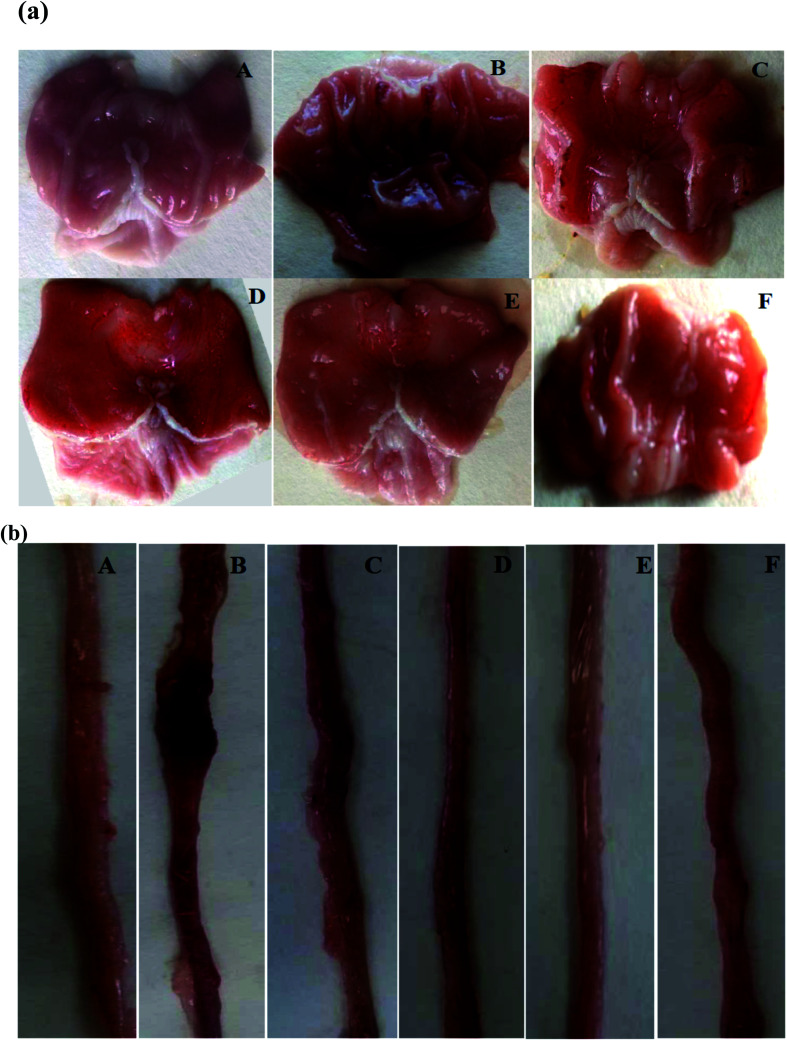
Gastric (a) and duodenal (b) morphology showing the protective effects of *Salvia officinalis* flowers decoction extract (SOFDE), mixture (MIX) and sulfasalazine (SULF) on EtOH-induced ulcer. Animals were treated with two doses of SOFDE (100 and 200 mg kg^−1^, p.o.), MIX (SOFDE, 50 mg kg^−1^, b.w., p.o. + SULF, 50 mg kg^−1^, b.w., p.o.), SULF (100 mg kg^−1^, p.o.) or vehicle (H_2_O). (A) H_2_O + NaCl; (B) H_2_O + EtOH; (C, D) SOFDE (100 and 200 mg kg^−1^, p.o., respectively) + EtOH; (E) MIX (SOFDE, 50 mg kg^−1^, b.w., p.o. + SULF 50 mg kg^−1^, b.w., p.o.) + EtOH; (F) sulfasalazine (100 mg kg^−1^, b.w., p.o.) + EtOH.

Macroscopic examination of the glandular part of the stomach and small intestine was performed at the opening of the gastrointestinal segments. EtOH-treatment induced hemorrhagic lesions on the glandular part of the stomach and along the duodenum (Fig. 2). However, SOFDE, MIX and sulfasalazine treatments significantly protected gastric and duodenal mucosa from alcohol-induced injury (Table 3).

#### Histopathological evaluation of gastric and duodenal lesions

**Fig. 3:**
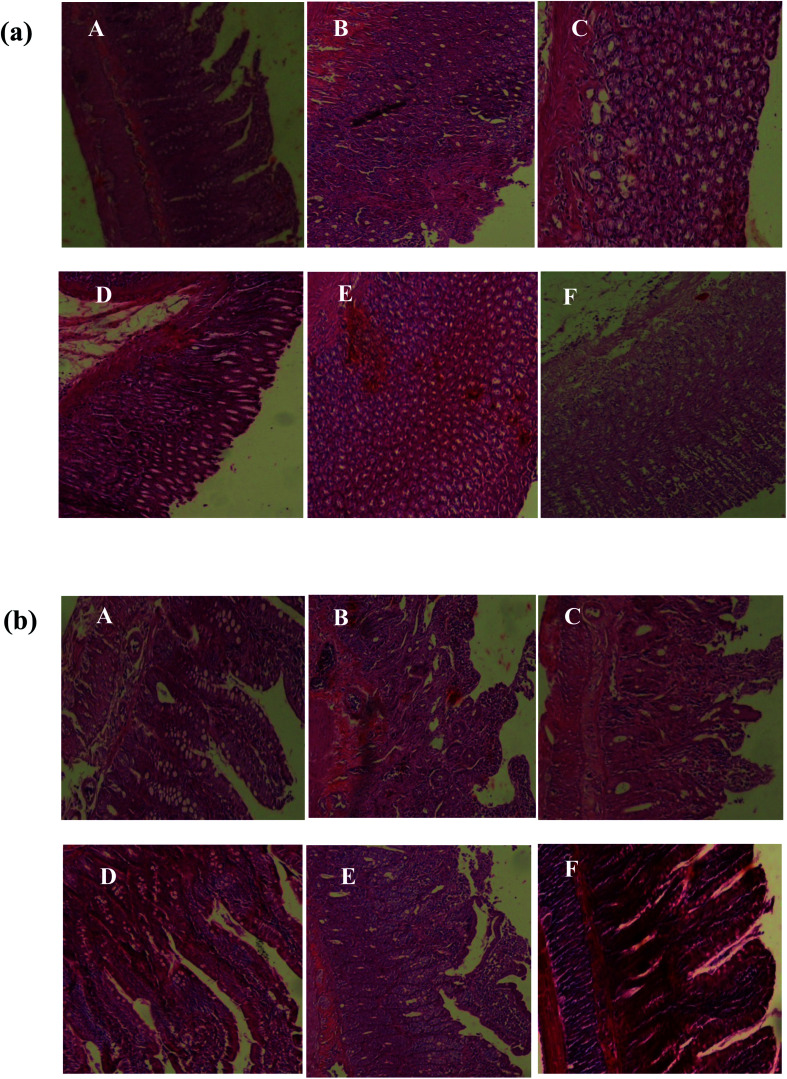
Gastric (a) and duodenal (b) histology showing the protective effects of *Salvia officinalis* flowers decoction extract (SOFDE), mixture (MIX) and sulfasalazine (SULF) on EtOH-induced ulcer. Animals were treated with two doses of SOFDE (100 and 200 mg kg^−1^, p.o.), MIX (SOFDE, 50 mg kg^−1^, b.w., p.o. + SULF, 50 mg kg^−1^, b.w., p.o.), SULF (100 mg kg^−1^, b.w., p.o.) or vehicle (H_2_O). (A) H_2_O + NaCl; (B) H_2_O + EtOH; (C, D) SFDE (100 and 200 mg kg^−1^, p.o., respectively) + EtOH; (E) MIX (SOFDE, 50 mg kg^−1^, b.w., p.o. + SULF 50 mg kg^−1^, b.w., p.o.) + EtOH; (F) sulfasalazine (100 mg kg^−1^, b.w., p.o.) + EtOH.

Histological observation of ethanol-induced gastric and intestinal lesions in EtOH group revealed a comparative extensive congestion, surface coating alteration, edema, necrotic lesions, epithelial and vascular cells alteration. We also observed a haemorrhage, hyperaemia as well as inflammatory cell infiltration in the stomach and small bowell mucosa as well as submucosa (Fig. 3).

Pretreatment with SOFDE showed a dose-dependent protection of the gastric and intestinal mucosa as revealed by the reduction of lesions, mucosal and submucosal edema as well as leucocytes infiltration. Importantly, we showed that the group received the mixture registered the most important protection. A similar protective effect had also observed in sulfasalazine pretreated rats.

#### Effect of SOFDE, sulfasalazine and mixture on EtOH-induced gastroduodenal lipoperoxidation and hydrogen peroxide increase

**Fig. 4:**
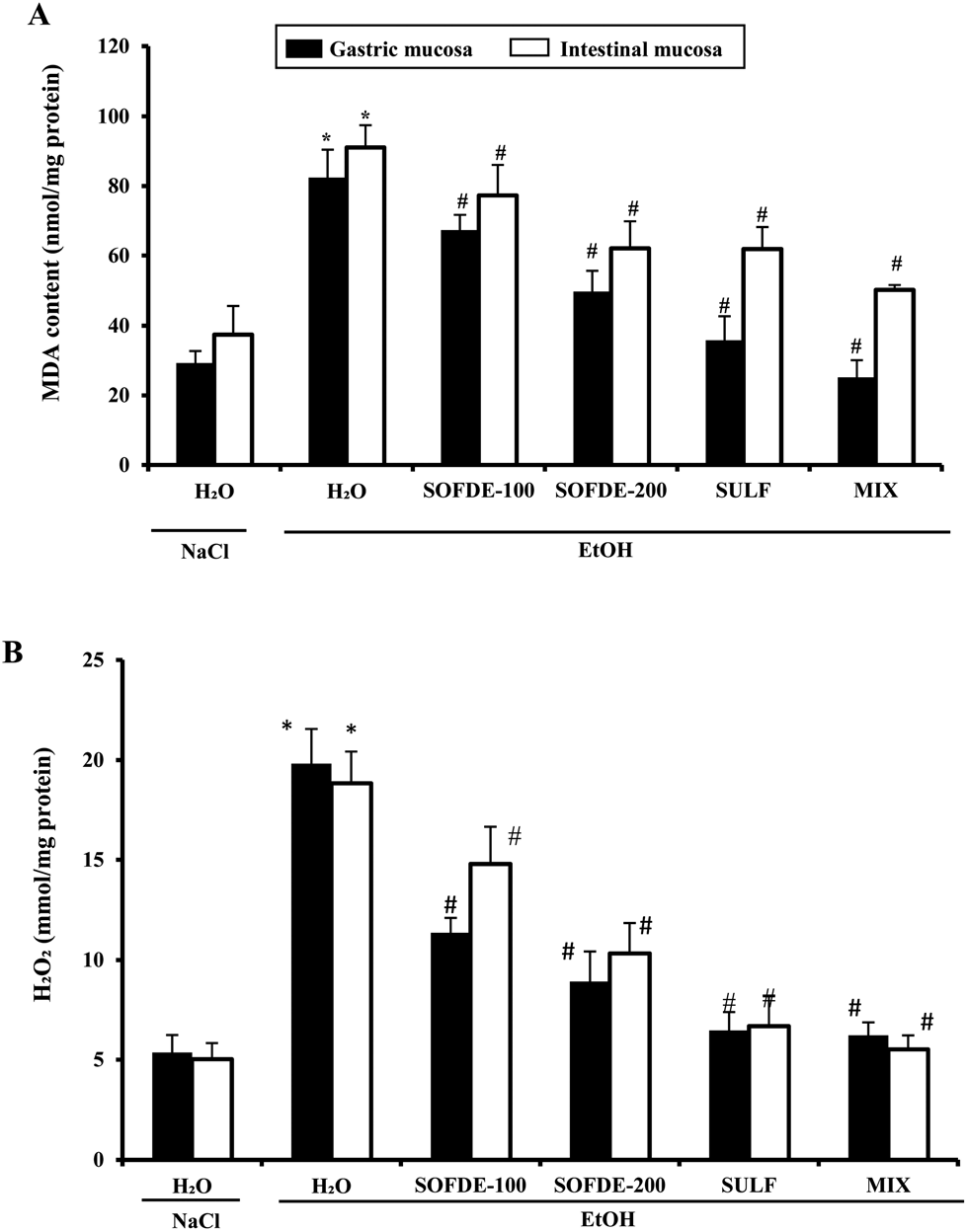
Effect of *Salvia officinalis* flowers decoction extract (SOFDE), mixture (MIX) and sulfasalazine (SULF) on EtOH-induced changes in stomach and intestinal mucosa MDA (A) and H_2_O_2_ (B) levels. Animals were pre-treated with two doses of SOFDE (100 and 200 mg kg^−1^, b.w., p.o.), SULF (100 mg kg^−1^, b.w., p.o.) and MIX (SOFDE, 50 mg kg^−1^, b.w., p.o. + SULF, 50 mg kg^−1^, b.w., p.o.) or vehicle (NaCl 0.9%). One hour after, animals received EtOH (4 g kg^−1^, b.w., p.o.) by gavage for 2 h. Assays were carried out in triplicate. *: *p* < 0.05 compared to control group and #: *p* < 0.05 compared to EtOH group.

To investigate the implication of oxidative stress in the antiulcerogenic effect of SOFDE, we firstly assessed the MDA and hydrogen peroxide levels. EtOH administration significantly increased MDA levels in gastric and duodenal mucosa. Alcohol-induced lipoperoxidation was significantly reversed by SOFDE, MIX or sulfasalazine pre-treatment in a dose-dependent manner (Fig. 4).

We also showed the effect of EtOH and SOFDE on intracellular mediator such as hydrogen peroxide level in gastric and intestinal mucosa (Fig. 4). In addition, alcohol group had a significant increase in hydrogen peroxide level in gastric and intestinal tissues when compared to negative control group. SOFDE and sulfasalazine treatment significantly and dose-dependently reduced the EtOH-induced this intracellular mediator deregulation. More importantly, our result found that MDA and H_2_O_2_ levels were reversed by MIX pre-treatment more significantly than sulfasalazine and SOFDE each alone.

#### Effects on plasma scavenging activity

**Fig. 5:**
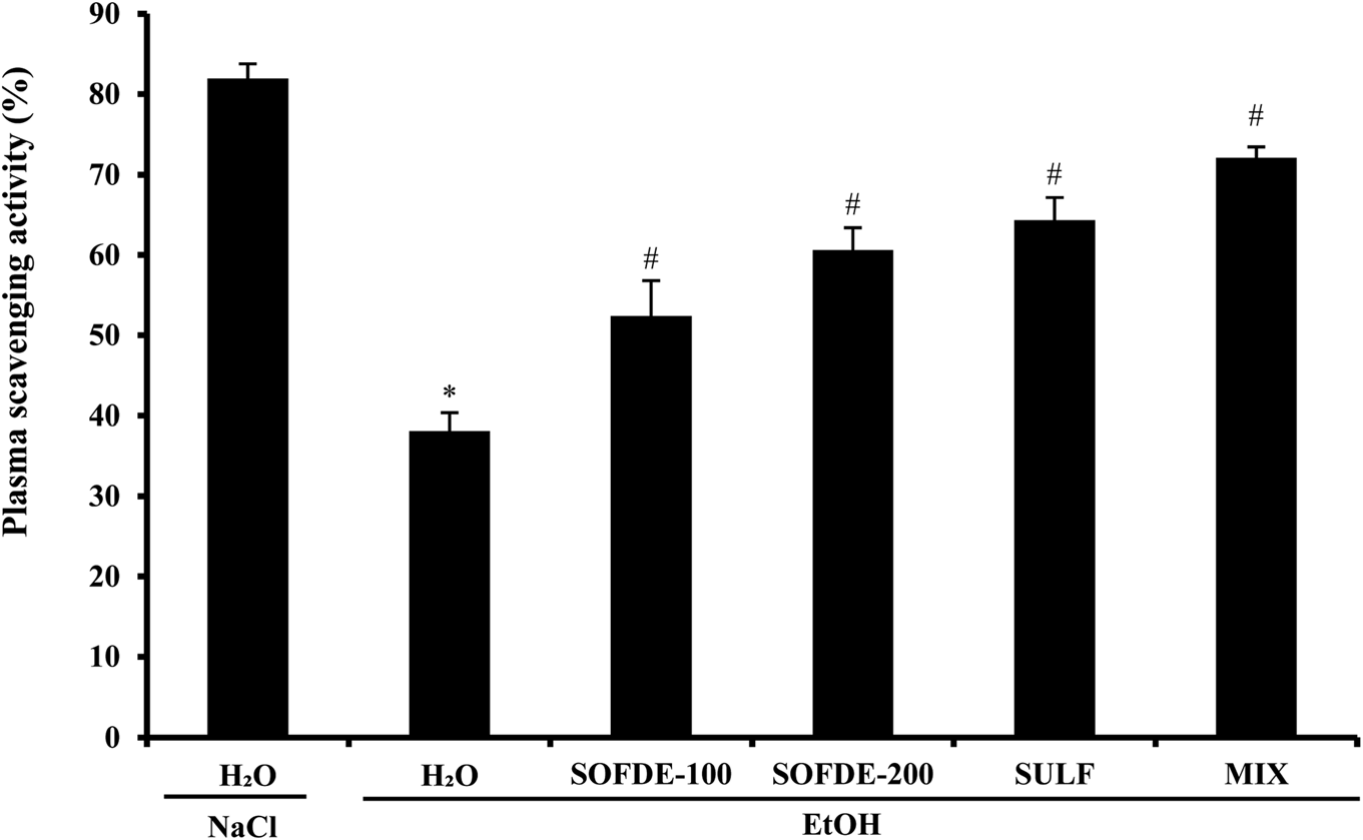
Effect of *Salvia officinalis* flowers decoction extract (SOFDE), mixture (MIX) and sulfasalazine (SULF) on EtOH-induced disturbances in plasma scavenging activity (PSA). Animals were pre-treated with two doses of SOFDE (100 and 200 mg kg^−1^, b.w., p.o.), SULF (100 mg kg^−1^, b.w., p.o.) and MIX (SOFDE, 50 mg kg^−1^, b.w., p.o. + SULF, 50 mg kg^−1^, b.w., p.o.) or vehicle (NaCl 0.9%). One hour after, animals received EtOH (4 g kg^−1^, b.w., p.o.) by gavage for 2 h. Assays were carried out in triplicate. *: *p* < 0.05 compared to control group and #: *p* < 0.05 compared to EtOH group.

EtOH administration significantly decreased the plasma scavenging activity when compared to control group (Fig. 5). By contrast, PSA percentage was significantly and dose-dependently increased after SOFDE pre-treatment. A similar effect was also observed for sulfasalazine, used as reference molecules, but less important than the group treated with the mixture.

#### Effect of SOFDE, SULF and MIX on EtOH-induced antioxidant enzyme activities depletion

**Fig. 6:**
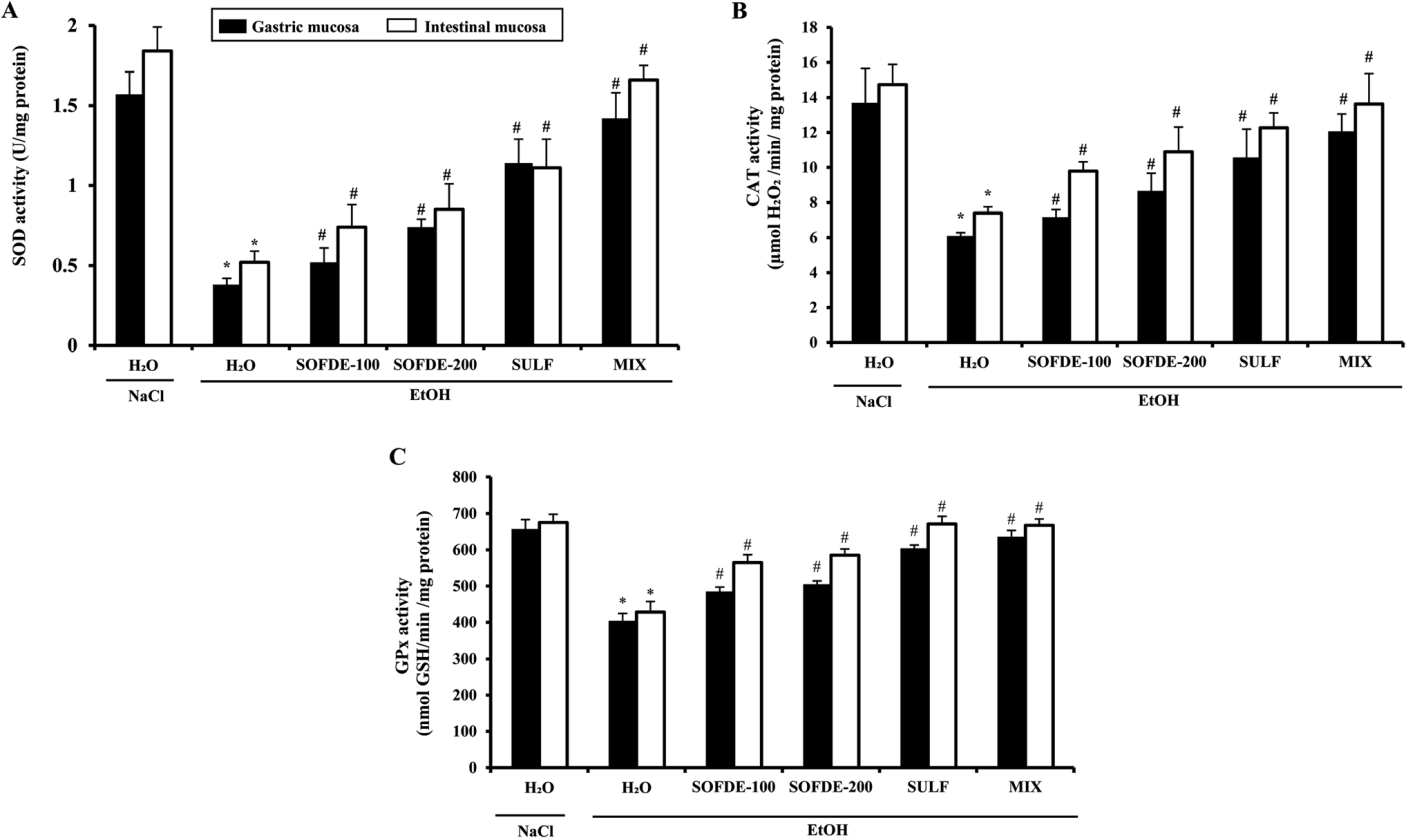
Effect of *Salvia officinalis* flowers decoction extract (SOFDE), mixture (MIX) and sulfasalazine (SULF) on EtOH-induced changes in stomach and intestinal mucosa antioxidant enzyme activities: SOD (A), CAT (B) and GPx (C). Animals were pre-treated with two doses of SOFDE (100 and 200 mg kg^−1^, b.w., p.o.), SULF (100 mg kg^−1^, b.w., p.o.) and MIX (SOFDE, 50 mg kg^−1^, b.w., p.o. + SULF, 50 mg kg^−1^, b.w., p.o.) or vehicle (NaCl 0.9%). One hour after, animals received EtOH (4 g kg^−1^, b.w., p.o.) by gavage for 2 h. Assays were carried out in triplicate. *: *p* < 0.05 compared to control group and #: *p* < 0.05 compared to EtOH group.

On other hand, we examined the effect of SOFDE, SULF and EtOH treatment on antioxidant enzyme activities (Fig. 6). As expected, gastric and duodenum injuries were accompanied by a significant decrease of superoxide dismutase (A), catalase (B) and glutathione peroxidase (C) activities. Sage decoction extract treatment significantly corrected the enzyme activities decrease caused by alcohol administration in a dose-related manner. The MIX exerted a more important effect than SOFDE and sulfasalazine each alone.

#### Effect of SOFDE, SUL and MIX on EtOH-induced non-enzymatic antioxidants levels deregulation

**Fig. 7:**
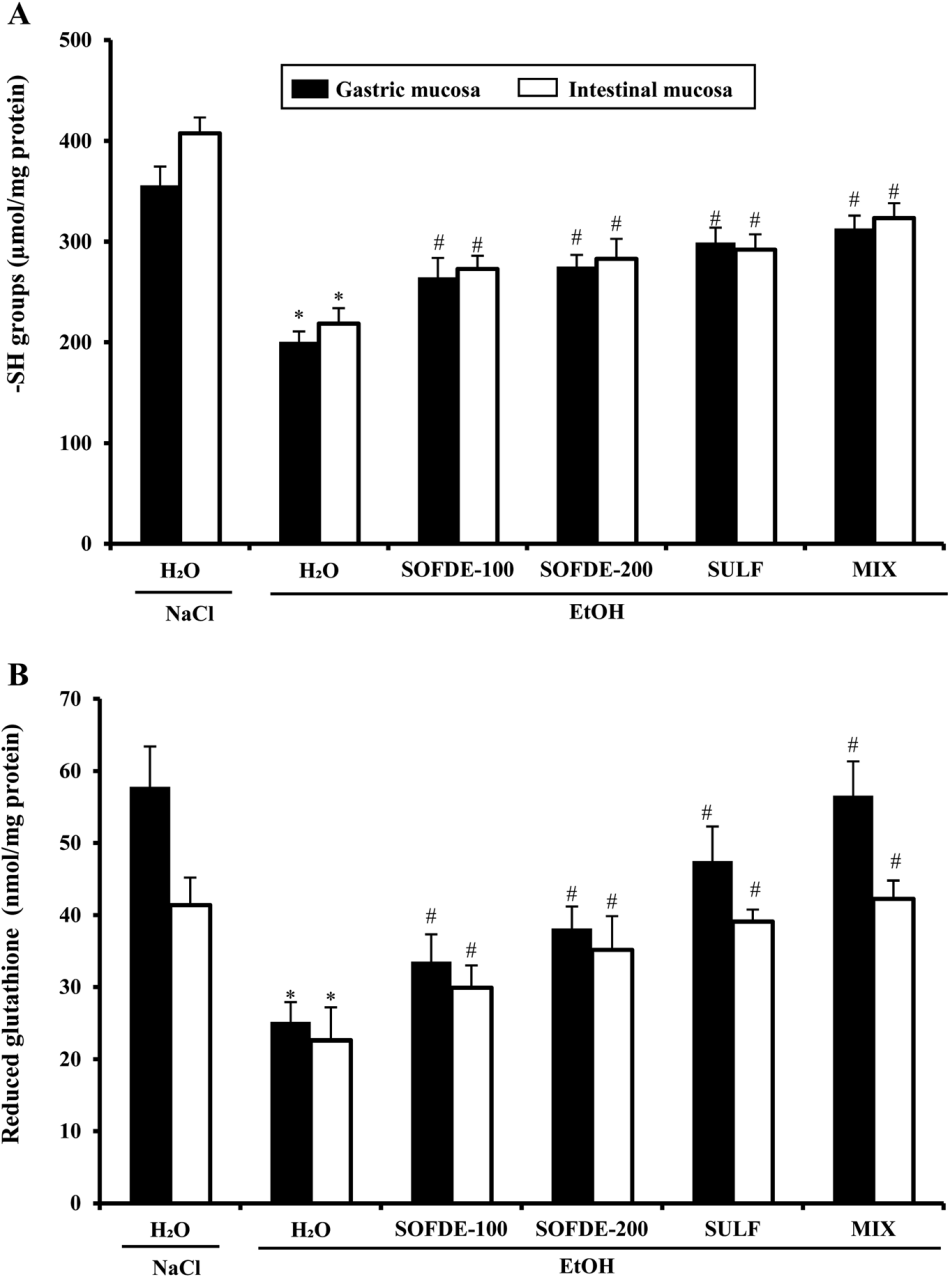
Effect of *Salvia officinalis* flowers decoction extract (SOFDE), mixture (MIX) and sulfasalazine (SULF) on EtOH-induced changes in stomach and intestinal mucosa sulfhydryl groups (A) and reduced glutathione (B). Animals were pre-treated with two doses of SOFDE (100 and 200 mg kg^−1^, b.w., p.o.), SULF (100 mg kg^−1^, b.w., p.o.) and MIX (SOFDE, 50 mg kg^−1^, b.w., p.o. + SULF, 50 mg kg^−1^, b.w., p.o.) or vehicle (NaCl 0.9%). One hour after, animals received EtOH (4 g kg^−1^, b.w., p.o.) by gavage for 2 h. Assays were carried out in triplicate. *: *p* < 0.05 compared to control group and #: *p* < 0.05 compared to EtOH group.

We also investigated the gastric and duodenal non-enzymatic antioxidants levels (Fig. 7). We showed that alcohol intoxication significantly reduced thiol groups (A) as well as reduced glutathione (B) contents. However, SOFDE exhibited significant and dose-dependent regulation of all those parameters. We noticed that the MIX exerts a more important effect than sulfasalazine alone.

#### Effect of SOFDE, SUL and MIX on free iron, magnesium and calcium levels

We further looked at the EtOH and SOFDE on intracellular mediators such as calcium, free iron and magnesium levels (Table 5). As expected, alcohol group showed a significant increase of those parameters in gastric and duodenal mucosa when compared to negative control group. SOFDE and SULF each alone significantly protected against EtOH-induced intracellular mediators disturbances while the mixture exerted a more pronounced effect.

**Table 5:** Subacute effect of *Salvia officinalis* flowers decoction extract (SOFDE), mixture (MIX) and sulfasalazine (SULF) on EtOH-induced changes in stomach and intestinal mucosa and plasma free iron, magnesium and calcium levels in rats. Animals were pre-treated with two doses of SOFDE (100 and 200 mg kg^−1^, b.w., p.o.), mixture (SOFDE, 50 mg kg^−1^, b.w., p.o. + SULF, 50 mg kg^−1^, b.w., p.o.) and SULF (100 mg kg^−1^, b.w., p.o.) or vehicle (NaCl 0.9%), and were challenged with a single oral administration of EtOH (4 g kg^−1^, b.w., p.o.) or NaCl (0.9%) for two hours. Assays were carried out in triplicate^a^

Groups	Free iron			Calcium			Magnesium		
	Gastric mucosa (μmol per mg per protein)	Intestinal mucosa (μmol per mg per protein)	Plasma (μmol L^−1^)	Gastric mucosa (mmol per mg per protein)	Intestinal mucosa (mmol per mg per protein)	Plasma (mmol L^−1^)	Gastric mucosa (μmol per mg per protein)	Intestinal mucosa (μmol per mg per protein)	Plasma (μmol L^−1^)
Control	22.02 ± 1.92	7.37 ± 1.05	1.28 ± 0.06	0.66 ± 0.08	0.73 ± 0.08	0.501 ± 0.04	194.32 ± 20.78	209.27 ± 19.69	24.30 ± 2.60
EtOH	63.09 ± 4.07*	20.18 ± 1.78*	2.26 ± 0.21*	2.57 ± 0.32*	3.11 ± 0.85*	0.99 ± 0.08*	364.63 ± 17.87*	334.46 ± 17.12*	98.66 ± 4.20*
EtOH + SOFDE-100	50.01 ± 3.90#	17.37 ± 1.40#	2.02 ± 0.13#	2.48 ± 0.78#	2.70 ± 0.58#	0.86 ± 0,07#	297.09 ± 19.74#	289.61 ± 18.21#	84.57 ± 4.09#
EtOH + SOFDE-200	44.22 ± 1.41#	15.62 ± 1.33#	1.69 ± 0.09#	1.87 ± 0.54#	2.26 ± 0.30#	0.71 ± 0.05#	266.26 ± 19.01#	263.46 ± 20.21#	63.70 ± 3.44#
EtOH + SULF	34.13 ± 3.87#	12.99 ± 2.56#	1.42 ± 0.15#	2.08 ± 0.79#	2.08 ± 0.78#	0.77 ± 0.06#	231.69 ± 22.61#	244.77 ± 21.22#	53.22 ± 5.33#
EtOH + MIX	30.45 ± 3.39#	8.42 ± 1.69#	1.31 ± 0.08#	1.44 ± 0.41#	1.31 ± 0.35#	0.55 ± 0.07#	200.86 ± 21.10#	216.74 ± 17.12#	49.51 ± 3.67#

a *: *p* < 0.05 compared to control group and ^#^: *p* < 0.05 compared to EtOH group.

#### Effect of SOFDE, SUL and MIX on EtOH-induced inflammation

Serum CRP and ALP activities significantly increased in the EtOH treated group when compared to control. Importantly, we found a protective effect against the inflammatory markers increase were observed in the SOFDE and SULF groups. We also showed a powerful anti-inflammatory activity of SOFDE against harmful effects of EtOH (Table 6). More importantly, we showed that the MIX presented a more important anti-inflammatory capacity when compared to SOFDE and SULF treatment each alone.

**Table 6:** Subacute effect of *Salvia officinalis* flowers decoction extract (SOFDE), mixture (MIX) and sulfasalazine (SULF) on EtOH-induced changes in plasma CRP and ALP levels in rats. Animals were pre-treated with two doses of SOFDE (100 and 200 mg kg^−1^, b.w., p.o.), mixture (SOFDE, 50 mg kg^−1^, b.w., p.o. + SULF, 50 mg kg^−1^, b.w., p.o.) and SULF (100 mg kg^−1^, b.w., p.o.) or vehicle (NaCl 0.9%), and were challenged with a single oral administration of EtOH (4 g kg^−1^, b.w., p.o.) or NaCl (0.9%) for two hours. Assays were carried out in triplicate^a^

Groups	C-Reactive protein (CRP) (μg dL^−1^)	Alkaline phosphatase (ALP) plasma (U L^−1^) at 37 °C
Control	0.34 ± 0.01	68.29 ± 3.72
EtOH	1.48 ± 0.37*	190.44 ± 5.04*
EtOH + SLAE-100	0.92 ± 0.21#	161.33 ± 1.67#
EtOH + SLAE-200	0.79 ± 0.12#	105.42 ± 4.30#
EtOH + SULF	0.69 ± 0.08#	83.19 ± 5.86#
EtOH + MIX	0.46 ± 0.01#	71.27 ± 8.84#

a *: *p* < 0.05 compared to control group and ^#^: *p* < 0.05 compared to EtOH group.

## Discussion

In the present work, we investigated the phytochemical properties as well as the individual or synergistic protective actions of *Salvia officinalis* flowers decoction extract and sulfasalazine on EtOH-induced peptic ulcer.

The phytochemical study firstly showed that SOFDE presents an important antioxidant activity assessed by to the bleaching inhibition potency of β-carotene (IC_50_ = 56.77 ± 2.34 μg mL^−1^). A similar result was obtained by Martins *et al.*^49^ who observed a good β-carotene bleaching inhibition (IC_50_ = 50.87 ± 3.73 μg mL^−1^). The antioxidant activity of SOFDE could be, in part, attributed to its high phenolic compounds levels. In this context, our data also suggest that SOFDE presents a high concentration of flavonols, total tannins and a moderate concentration of total anthocyanins and carotenoids. These levels fully corroborated those of Akhondzadeh *et al.*^22^ The antioxidant capacity is mainly attributed to the hight level of phenolic acids and flavonoids in this fraction such as quinic, protocatchuic, 1,3-di-*O*-caffeoyquinic, *p*-coumaric and salviolinic acids, and naringin, quercetin, kampherol, apigenin-7-*o*-glucoside, luteolin-7-*o*-glucoside and cirsilineol. It has been previously found that the majority of these bioactive molecules has been identified in aqueous leaf extracts and has been shown for a high antioxidant capacity against DPPH radical.^32^

*In vivo*, we firstly revealed that acute alcohol administration distorted the gastric and duodenal mucosa and submucosa, which are accompanied by surface coating, epithelial cells alterations, as well as edema and leukocyte infiltration. Indeed, prostaglandin deficiency within the digestive mucosa is defined as the major pathogenic mechanism of the ethanol-induced digestive system diseases. The deficit in endogenous prostaglandins plays an essential role in the pathogenic process by making the mucosa more vulnerable to the aggression, without direct implication in the digestive lesions.^50^ Our data are in line with previous report using EtOH as ulceration inducer.^51^ Ethanol causes injures in the vascular endothelial cells of the gastric and intestine mucosa and induces microcirculatory disturbance and hypoxia, related to massive production of free radicals.^52^

Importantly, our data showed a protective effect of subaccute treatment with SOFDE (15 days) against gastric and intestinal fluid accumulation, weight of mucus and lesions induced by EtOH administration. Our extract also contributed in the reduction of macroscopic and histopathological observed damages. The therapeutic effect of MIX is more pronounced than SULF (100 mg kg^−1^, b.w., p.o.) used as reference molecule as well as the high dose of SOFDE (200 mg kg^−1^, b.w., p.o.). This beneficial effect can be explained by the fact that this drug has a single course of action^53^ while sage decoction extract acts through several different mechanisms.^54^ When those two mechanisms are combined in the gastrointestinal tract, synergism will occur through several biochemical targets and pathways. However, for phenolic compounds many mechanisms might be involved such as intracellular mediators by chelation of metal ions (Fe^2+^, Cu^2+^), membrane stabilization,^55^ inhibition of pepsinogen production^56^ and increased mucus production, characterized by a film formed by the polymerization of glycoproteins which makes it possible to trap the bicarbonates and delay the penetration of endoluminous H^+^ ions. This situation establish a pH gradient ranging from less than 3 at the level of the luminal face of this layer, to more than 7 on the mucosal surface.^57^ However, the mixture of natural active substances and drugs has been used in previous work to treat cancer diseases^58^ as well as its antibacterial activity.^59^

In the present study, we also showed a high concentration of total tannins (60.41 ± 3.87 mg TAE/g DM). These molecules could prevent ulcer development throughout vasoconstricting effects or to their proteins precipitation in the ulceration site, producing an impermeable coating over the lining that restrains gut secretions and defends the underlying mucosa from lesions.^60^ We also found a high level of flavonls (kaempferol and quercetin), that present anti-ulcer and gastroprotective properties.^61^

We also showed that EtOH intoxication induced a depletion of plasma scavenging activity (PSA), as well as an increase of hydrogen peroxide and MDA level, which is defined as an indicator of the ROS generation in tissues and plasma. In addition, we observed a decrease of thiol group and GSH levels, as well as depletion antioxidant enzyme activities such SOD, CAT and GPx. However, its well known that acute administration of EtOH leads oxidative imbalance through several pathways such as the generation of reactive oxygen species.^62^ Indeed, SOD converts the reactive superoxide radical to H_2_O_2_, which was decreased in the gastric and intestine mucosa. When this intracellular mediator was not scavenged by CAT, it could leads to lipid peroxidation after generation of hydroxyl radical.^63^ More importantly, we showed that SOFDE and sulfasalazine administration each alone or in combination (MIX) abolished acute EtOH-induced oxidative stress in the gastric and the duodenal mucosa. These finding are similar with previous study which has reported that the sage decoction extract contains a good amount of total polyphenols, flavonoïds, condensed tannins and a high level of rosmarinic and salviolinic acids and apegenin-7-*O*-glucoside.^49^ These molecules are the primal source of the antioxidant ability of this plant and are including in the scavenging of free radicals as hydroxyl radical (OH˙) which is the major cause of lipid peroxidation.^64^ Furthermore, those sulfhydryl groups are in part involved in gastric cytoprotection^65^ as well as in the maintain of mucosal barrier integrity and free radicals scavenging.

Importantly, we showed an increase of intracellular mediators such as calcium, free iron and magnesium in plasma, gastric and duodenal mucosa in response to oxidative stress induced by ethanol administration. These data are in line with several previous studies.^66,67^ However, we can now suggest that SOFDE exerts a beneficial effect by chelating free iron and scavenging H_2_O_2_ and regulation of the calcium and magnesium homeostasis. Our results also supposes that pretreatment with SOFDE protects against overcharge of cells of the gastric and intestinal mucosa by free iron and H_2_O_2_ induced by ethanol sub-acute administration. Moreover, these later are involved in the generation of hydroxyl radical (OH˙),^68^ which plays the major role in oxidative damage by affecting the molecular structures.^69^ In this respect, Jan *et al.*^70^ conclude that living organisms create a complex endogenous and exogenous antioxidant defense system to restrict the production of this damaging radical.

Finally, we have shown in the present work that EtOH intoxication induced inflammation as assessed by a significant increase in plasma CRP and ALP when compared to control group (*P* < 0.05). On the other hand, the pre-treatment with SOFDE, sulfasalazine and MIX significantly decreased the studied biomarkers. These data are in line with those of Mosli *et al.*^71^ Indeed, several studies have shown that EtOH is associated with an inflammatory state *via* the expression of pro-inflammatory cytokines.^51^ However, oxidative stress is well known to be related to inflammatory gastric and bowel disease.^72^ Moreover, quinic, salviolinic, procatchuic and *p*-coumaric acids which are identified with abandoned amount in SOFDE possess an important anti-inflammatory activity.^29,73^

## Conclusion

In conclusion, our data clearly demonstrated strong synergic protective effects between *Salvia officinalis* decoction extract and sulfasalazine against ethanol-induced gastric and small bowel injuries. This gastro-duodenal protection might be due in part to its antioxidant and anti-inflammatory properties as well as its opposite effects on intracellular mediators such as hydrogen peroxide, free iron and calcium. Therefore, the mixture between bioactive compounds from plant and standards drugs is considered as an alternative to protect against gastro-intestinal disorders and to avoid unpredictable side effects of commercial drugs.

## Ethical consideration

All procedures on animals in this study were approved with the National Institute of Health recommendations for the use and care of animals.

## Abbreviations

CAT: CAT
EtOH: Ethanol
GPx: Glutathione peroxidase
GSH: Reduced glutathione
H_2_O_2_: Hydrogen peroxide
MDA: Malondialdehyde
MIX: Mixture
ROS: Reactive oxygen species
–SH: Sulfhydryl groups
SOD: Superoxide dismutase
SOFDE: *Salvia officinalis* flowers decoction extract
SULF: Sulfasalazine

## Conflicts of interest

There are no conflicts to declare.

## Supplementary Material

## References

[cit1] Dong S. X. M., Chang C. C. Y., Rowe K. J. (2018). A collection of the etiological theories, characteristics, and observations/phenomena of peptic ulcers in existing data. Data Brief.

[cit2] Suzuki H., Iwasaki E., Hibi T. (2009). *Helicobacter pylori* and gastric cancer. Gastric Cancer.

[cit3] Vonkeman H. E., Klok R. M., Postma M. J., Brouwers J. R., van de Laar M. A. (2007). Direct medical costs of serious gastrointestinal ulcers among users of NSAIDs. Drugs Aging.

[cit4] Maity P., Biswas K., Roy S., Banerjee R. K., Bandyopadhyay U. (2003). Nov Smoking and the pathogenesis of gastroduodenal ulcer recent mechanistic update. Mol. Cell. Biochem..

[cit5] Ceglia L., Harris S. S., Rasmussen H. M., Dawson-Hughes B. (2009). Activation of the calcium sensing receptor stimulates gastrin and gastric acid secretion in healthy participants. Osteoporosis Int..

[cit6] Bujanda L. (2000). The effects of alcohol consumption upon the gastrointestinal tract. Am. J. Gastroenterol..

[cit7] Sun J., Fu J., Zhong Y., Li L., Chen C., Wang X., Wang L., Hou Y., Wang H., Zhao R., Zhang X., Yamamoto M., Xu Y., Pi J. (2018). NRF2 mitigates acute alcohol-induced hepatic and pancreatic injury in mice. Food Chem. Toxicol..

[cit8] Hamaguchi M., Watanabe T., Higuchi K., Tominaga K., Fujiwara Y., Arakawa T. (2001). Mechanisms and roles of neutrophil infiltration in stress-induced gastric injury in rats. Dig. Dis. Sci..

[cit9] Grüning N. M., Rinnerthaler M., Bluemlein K., Mülleder M., Wamelink M. M., Lehrach H., Jakobs C., Breitenbach M., Ralser M. (2011). Pyruvate kinase triggers a metabolic feedback loop that controls redox metabolism in respiring cells. Cell Metab..

[cit10] Halliwell B. (2007). Biochemistry of oxidative stress. Biochem. Soc. Trans..

[cit11] Almroth B. C., Sturve J., Berglund A., Förlin L. (2005). Oxidative damage in eelpout (*Zoarces viviparus*), measured as protein carbonyls and TBARS, as biomarkers. Aquat. Toxicol..

[cit12] Litvin T. N., Kamenetsky M., Zarifyan A., Buck J., Levin L. R. (2003). Kinetic properties of "soluble" adenylyl cyclase. Synergism between calcium and bicarbonate. J. Biol. Chem..

[cit13] Tashiro N., Budhathoki S., Ohnaka K., Toyomura K., Kono S., Ueki T., Tanaka M., Kakeji Y., Maehara Y., Okamura T., Ikejiri K., Futami K., Maekawa T., Yasunami Y., Takenaka K., Ichimiya H., Terasaka R. (2011). Constipation and colorectal cancer risk: the Fukuoka Colorectal Cancer Study. Asian Pac. J. Cancer Prev..

[cit14] Barbero P., Liascovich R., Valdez R., Moresco A. (2011). Misoprostol teratogenicity: a prospective study in Argentina. Arch. Argent. Pediatr..

[cit15] Lindberg B. U., Broomé U., Persson B. (2001). Proximal colorectal dysplasia or cancer in ulcerative colitis. The impact of primary sclerosing cholangitis and sulfasalazine: results from a 20-year surveillance study. Dis. Colon Rectum.

[cit16] Pachajoa H., Isaza C. (2011). First case of Moebius-Poland syndrome in child prenatally exposed to misoprostol. Neurol Barc Spain.

[cit17] Jedidi S., Rtibi K., Selmi S., Aloui F., Selmi H., Wannes D., Sammari H., Dhawefi N., Abbes C., Sebai H. (2019). Phytochemical/antioxidant properties and individual/synergistic actions of *Salvia officinalis* L. aqueous extract and loperamide on gastrointestinal altering motor function. J. Med. Food.

[cit18] Walker J. B., Sytsma K. J., Treutlein J., Wink M. (2004). *Salvia* (Lamiaceae) is not monophyletic: implications for the systematics, radiation, and ecological specializations of Salvia and tribe Mentheae. Am. J. Bot..

[cit19] Rasmy N. M., Hassan A. A. (2012). Foda MI and El-Moghazy MM Assessment of the antioxidant activity of sage (*Salvia officinalis* L.) extracts on the shelf life of mayonnaise. World J. Dairy Food Sci..

[cit20] Fawzi M., Kamel Z., Farhan S. (2017). Anti-inflammatory effect of sage (*Salvia officinalis*) extracts abstract on oral health. Iraqi Dent. J..

[cit21] Monsefi M., Abedian M., Azarbahram Z., Ashraf M. J. (2015). *Salvia officinalis* L. induces alveolar bud growing in adult female rat mammary glands. Avicenna J. Phytomed..

[cit22] Akhondzadeh S., Noroozian M., Mohammadi M., Ohadinia S., Jamshidi A. H., Khani M. (2003). *Salvia officinalis* extract in the treatment of patients with mild to moderate Alzheimer's disease: a double blind, randomized and placebo-controlled trial. J. Clin. Pharm. Ther..

[cit23] Stanojevic D., Comic L., Stefanovic O., Solujic-Sukdolak S. (2010). *In vitro* synergistic antibacterial activity of *Salvia officinalis* and some preservatives. Arch. Biol. Sci..

[cit24] Jedinák A., Mucková M., Kost'álová D., Maliar T., Masterova I. (2006). Antiprotease and antimetastatic activity of ursolic acid isolated from *Salvia officinalis*. Z. Naturforsch., C: J. Biosci..

[cit25] Coon J. T., Ernst E. (2002). Systematic review: herbal medicinal products for non-ulcer dyspepsia. Aliment. Pharmacol. Ther..

[cit26] Wang M., Kikuzaki H., Zhu N., Sang S., Nakatani N., Ho C. T. (2000). Isolation and structural elucidation of two new glycosides from sage (*Salvia officinalis* L.). J. Agric. Food Chem..

[cit27] Badiee P., Nasirzadeh A. R., Motaffaf M. (2012). Comparison of *Salvia officinalis* L. essential oil and antifungal agents against candida species. J. Pharm. Technol. Drug Res..

[cit28] Mansourabadi A. M., Sadeghi H. M., Razavi N., Rezvani E. (2015). Anti-inflammatory and analgesic properties of salvigenin, Salvia officinalis flavonoid extracted. Adv. Herb. Med..

[cit29] Baricevic D., Sosa S., Della Loggia R., Tubaro A., Simonovska B., Krasna A., Zupancic A. (2001). Topical anti-inflammatory activity of *Salvia officinalis* L. leaves: the relevance of ursolic acid. J. Ethnopharmacol..

[cit30] Nicolella H., Senedese J., Furtado R., Veneziani R., Tavares D. (2015). Evaluation of antiinflammatory potential of diterpene manool in macrophages by quantification of nitric oxide. J. Int. Soc. Antioxid. Nutr. Health.

[cit31] AOAC , Official methods of analysis, ed. Association of Official Analytical Chemists, Washington DC, 1990

[cit32] Jedidi S., Selmi H., Aloui F., Rtibi K., Jridi M., Abbes C., Sebai H. (2019). Comparative studies of phytochemical screening, HPLC-PDA-ESIMS/MS-LC/HR-ESI-MS analysis, antioxidant capacity and *in vitro* fermentation of officinal sage (*Salvia officinalis* L.) cultivated in different biotopes of Northwestern Tunisia. Chem. Biodiversity.

[cit33] Kujala T. S., Loponen J. M., Klika K. D., Pihlaja K. (2000). Phenolics and betacyanins in red beetroot (*Beta vulgaris*) root: distribution and effect of cold storage on the content of total phenolics and three individual compounds. J. Agric. Food Chem..

[cit34] Rigane G., Ben Salem R., Sayadi S., Bouaziz M. (2011). Phenolic composition, isolation and structure of a new deoxyloganic acid derivative from Dhokar and Gemri-Dhokar olive cultivars. J. Food Sci..

[cit35] Marina I. H., Velumuttu O., Pekka E. K. (1989). Carotenoids in finish foods: vegetables, fruits and berries. J. Agric. Food Chem..

[cit36] Giusti M. M. and Wrolstad R. E., Anthocyanins: characterization and measurement with UV-visible spectroscopy Current protocols in food analytical chemistry banner, John Wiley & Sons, New York, 2001, pp. 19–32

[cit37] Kulisica T., Radonic A., Katalinic V., Milos M. (2004). Use of different methods for testing antioxidant activity of oregano essential oil. Food Chem..

[cit38] Rtibi K., Hammami I., Selmi S., Grami D., Sebai H., Amri M., Marzouki L. (2017). Phytochemical properties and pharmacological effects of *Quercus ilex* L. aqueous extract on gastrointestinal physiological parameters *in vitro* and *in vivo*. Biomed. Pharmacother..

[cit39] Dicarlo G. D., Mascolo N., Izzo A. A., Capasso F., Autore G. (1994). Effects of quercetin on gastrointestinal tract in rats and mice. Phytother. Res..

[cit40] Brand-Williams W., Cuvelier M. E., Berset C. (1995). Use of a free radical method to evaluate antioxidant activity. LWT--Food Sci. Technol..

[cit41] Draper H. H., Hadley M. (1990). Malondialdehyde determination as index of lipid peroxidation. Methods Enzymol..

[cit42] Dingeon B., Ferry J. P., Roullet A. (1975). Automatic assay of blood sugar by Trinder's method. Ann. Biol. Clin..

[cit43] Kakkar P., Das B., Viswanathan P. N. (1984). Modified spectrophotometric assay of SOD. Indian J. Biochem. Biophys..

[cit44] Aebi H. (1984). Catalase *in vitro*. Methods Enzymol..

[cit45] Flohé L., Gunzler W. A. (1984). Assays of glutathione peroxidase. Methods Enzymol..

[cit46] Ellman G. L. (1959). Tissue sulfhydryl groups. Arch. Biochem. Biophys..

[cit47] Sedlak J., Lindsay R. H. (1968). Estimation of total, protein-bound, and non-protein sulfhydryl groups in tissue with Ellman's reagent. Anal. Biochem..

[cit48] Hartree E. F. (1972). Determination of protein: a modification of the Lowry method that gives a linear photometric response. Anal. Biochem..

[cit49] Martins N., Barros L., Santos-Buelga C., Henriques M., Silva S., Ferreira I. C. (2015). Evaluation of bioactive properties and phenolic compounds in different extracts prepared from *Salvia officinalis* L. Food Chem..

[cit50] Amandeep K., Robin S., Ramica S., Sunil K. (2012). Peptic ulcer: a review on etiology and pathogenesis. Int. Res. J. Pharm..

[cit51] Selmi S., Rtibi K., Grami D., Sebai H., Marzouki L. (2017). Protective effects of orange (*Citrus sinensis* L.) peel aqueous extract and hesperidin on oxidative stress and peptic ulcer induced by alcohol in rat. Lipids Health Dis..

[cit52] Suzuki H., Nishizawa T., Sugawa H. T., Mogami S., Hibi T. (2015). Roles of oxidative stress in stomach disorders. J. Clin. Biochem. Nutr..

[cit53] Dale J., Alcorn N., Capell H., Madhok R. (2007). Combination therapy for rheumatoid arthritis: methotrexate and sulfasalazine together or with other DMARDs. Nat. Clin. Pract. Rheumatol..

[cit54] Procházková D., Boušová I., Wilhelmová N. (2011). Antioxidant and prooxidant properties of flavonoids. Fitoter.

[cit55] Czinner E., Hagymasi K., Blazovics A., Kery A., Szoke E., Lemberkovics E. (2001). The *in vitro* effect of *Helichysi flos* on microsomal lipid peroxidation. J. Ethnopharmacol..

[cit56] Alanko J., Riutta A., Holm P., Mucha I., Vapatalo H., Metsa-Ketela T. (1999). Modulation of arachidonic acid metabolism by phenols: relation to their structure and antioxidant/prooxidant properties. Free Radicals Biol. Med..

[cit57] Gufo Kamguia H. F., Fokunang C., Ngameni B., Njinkio Nono B., Tembe-Fokunang E. (2011). Effet cytoprotecteur de l’extrait aqueux des racines de *Dorstenia psilurus* sur l’ulcère gastrique chez les rats males de la souche Wistar. Health Sci. Dis..

[cit58] Zhou D. H., Wang X., Yang M., Shi X., Huang W., Feng Q. (2013). Combination of low concentration of (−)epigallocatechingallate (EGCG) and curcumin strongly suppresses the growth of non small cell lung cancer in vitro and in vivo through causing cell cycle arrest. Int. J. Mol. Sci..

[cit59] Bhardwaj M., Singh B. R., Sinha D. K., Kumar V., PrasannaVadhana O. R., Varan Singh S., Nirupama K. R., Shree P., Archana Saraf B. S. (2016). Potential of herbal drug and antibiotic combination therapy: a new approach to treat multidrug resistant bacteria. Pharmac. Anal. Acta.

[cit60] deJesus N. Z., de Souza Falcão H., Gomes I. F., de Almeida Leite T. J., de Morais Lima G. R., Barbosa-Filho J. M., Tavares J. F., da Silva M. S., de Athayde-Filho P. F., Batista L. M. (2012). Tannins, peptic ulcers and related mechanisms. Int. J. Mol. Sci..

[cit61] Hodek P., Trefil P., Stiborova M. (2002). Flavonoids-potent and versatile biologically active compounds interacting with cytochromes P450. Chem.-Biol. Interact..

[cit62] Pérez-Gallardo R. V., Briones L. S., Díaz-Pérez A. L., Gutiérrez S., Rodríguez-Zavala J. S., Campos-García J. (2013). Reactive oxygen species production induced by ethanol in *Saccharomyces cerevisiae* increases because of a dysfunctional mitochondrial iron-sulfur cluster assembly system. FEMS Yeast Res..

[cit63] Yin H., Xu L., Porter N. A. (2011). Free radical lipid peroxidation: mechanisms and analysis. Chem. Rev..

[cit64] Kadhim S. M., Mohammed M. T., Ahmed O. M., Jassim A. M. N. (2016). Study of some *Salvia officinalis* L. (sage) components and effect of their aqueous extract on antioxidant. Int. J. Chem. Sci..

[cit65] Natale G., Lazzeri G., Lubrano V., Colucci R., Vassalle C., Fornai M., Blandizzi C., delTacca M. (2004). Mechanisms of gastroprotection by lansoprazole pretreatment against experimentally induced injury in rats: role of mucosal oxidative damage and sulfhydryl compounds. Toxicol. Appl. Pharmacol..

[cit66] Günther T., Vormann J., McGuigan J. A. (1995). Buffering and activity coefficient of intracellular free magnesium concentration in human erythrocytes. Biochem. Mol. Biol. Int..

[cit67] Rainbow R. D., Macmillan D., McCarron J. G. (2009). The sarcoplasmic reticulum Ca2+ store arrangement in vascular smooth muscle. Cell Calcium.

[cit68] Bagryanskaya E. G., Krumkacheva O. A., Fedin M. V., Marque S. R. A. (2015). Development and application of spin traps, spin probes, and spin labels. Methods Enzymol..

[cit69] Willcox J. K., Ash S. L., Catignani G. L. (2004). Antioxidants and prevention of chronic disease. Crit. Rev. Food Sci. Nutr..

[cit70] Jan A. T., Azam M., Siddiqui K., Ali A., Choi I., Haq Q. M. R. (2015). Heavy metals and human health: mechanistic insight into toxicity and counter defense system of antioxidants. Int. J. Mol. Sci..

[cit71] Mosli M. H., Zou G., Garg S. K., Feagan S. G., MacDonald J. K., Chande N., Sandborn W. J., Feagan B. G. (2015). C-reactive protein, fecal calprotectin, and stool lactoferrin for detection of endoscopic activity in symptomatic inflammatory bowel disease patients: A systematic review and meta-analysis. Am. J. Gastroenterol..

[cit72] Balmus I. M., Ciobica A., Trifan A., Stanciu C. (2016). The implications of oxidative stress and antioxidant therapies in inflammatory bowel disease: clinical aspects and animal models. Saudi J. Gastroenterol..

[cit73] Lima C. F., Andrade P. B., Seabra R. M., Fernandes-Ferreira M., Pereira-Wilson C. (2005). The drinking of a *Salvia officinalis* infusion improves liver antioxidant status in mice and rats. J. Ethnopharmacol..

